# Ulcer in a Bulbar Diverticulum: An Atypical Localization and Rare Complication of Gastrointestinal Hemorrhage

**DOI:** 10.7759/cureus.74551

**Published:** 2024-11-27

**Authors:** Fatima Adamou, Morgane Faivre, Charline Briot

**Affiliations:** 1 Gastroenterology, Mohammed VI University Hospital, Oujda, MAR; 2 Gastroenterology, Groupe Hospitalier de la Haute Saone, Vesoul, FRA

**Keywords:** bleeding, diverticulum, gastro intestinal, gastroscopy, ulcer

## Abstract

Gastrointestinal bleeding remains a frequent reason for emergency consultations, with a mortality rate that is still worrying despite advances in treatment. The most common cause is gastro-duodenal ulcers, mainly linked to Helicobacter pylori. Unusual causes such as gastroduodenal diverticular haemorrhage, a rare and serious complication, can also be detected during endoscopy. Although duodenal diverticula are the second most common localization after the colon, gastric intra-diverticular ulcers are extremely rare and rarely described in the literature. Treatment of diverticular haemorrhage can range from medical therapy to diverticulotomy. We present a case of upper gastrointestinal bleeding due to an unusual localization, specifically a gastro-duodenal ulcer situated within a diverticulum of the bulb. This article emphasizes the importance of meticulous exploration of gastric diverticula during gastroscopy, as they may conceal ulcers. Such endoscopic interventions can significantly impact patient management and prognosis, potentially altering outcomes from simple proton pump inhibitor therapy to preventing mortality, which remains alarmingly high according to existing literature.

## Introduction

Upper gastrointestinal bleeding is a common reason for emergency admissions, with a global mortality rate ranging from 5% to 10% according to literature data [1]. This mortality rate was higher in men (10.22%) than women (8.25%) especially in acute gastrointestinal hemorrhage cases [1]. The average age was 66.8 years, ranging from 21 to 97 [2]. Risk factors for upper GI bleeding include medications like non-steroidal anti-inflammatory drugs (NSAIDs) and anticoagulants, as well as older age, alcohol, smoking, and obesity [3 ]. Ulcer pathology is the primary cause of this type of bleeding, followed by portal hypertension, gastro-duodenal erosions, and esophagitis, collectively accounting for a global prevalence of approximately 80% [2,3]. In France, the incidence of upper gastrointestinal bleeding, which has been decreasing, is currently around 120 per 100,000 inhabitants per year. Despite this decline, gastroduodenal ulcers remain the leading cause of upper gastrointestinal bleeding [4]. Diverticula (small sacs or hernias formed by folds in the mucous membrane of the intestinal wall or esophagus) are rarely found in the stomach but are present in the duodenum, where they rank as the second most common location after the colon. Their incidence in the general population is not well-defined, globally ranging from 2% to 5% [5], though it can reach up to 23% according to endoscopic or autopsy studies [6]. In 90% of cases, duodenal diverticula are asymptomatic [7]. An unusual etiology for upper gastrointestinal bleeding is duodenal diverticular hemorrhage [5]. In this report, we present a case of upper gastrointestinal bleeding due to an intra-diverticular ulcer located at the duodenal bulb. This atypical location, which is very difficult to detect and assess during gastroscopy, highlights the uniqueness of our article.

## Case presentation

**Figure 1:**
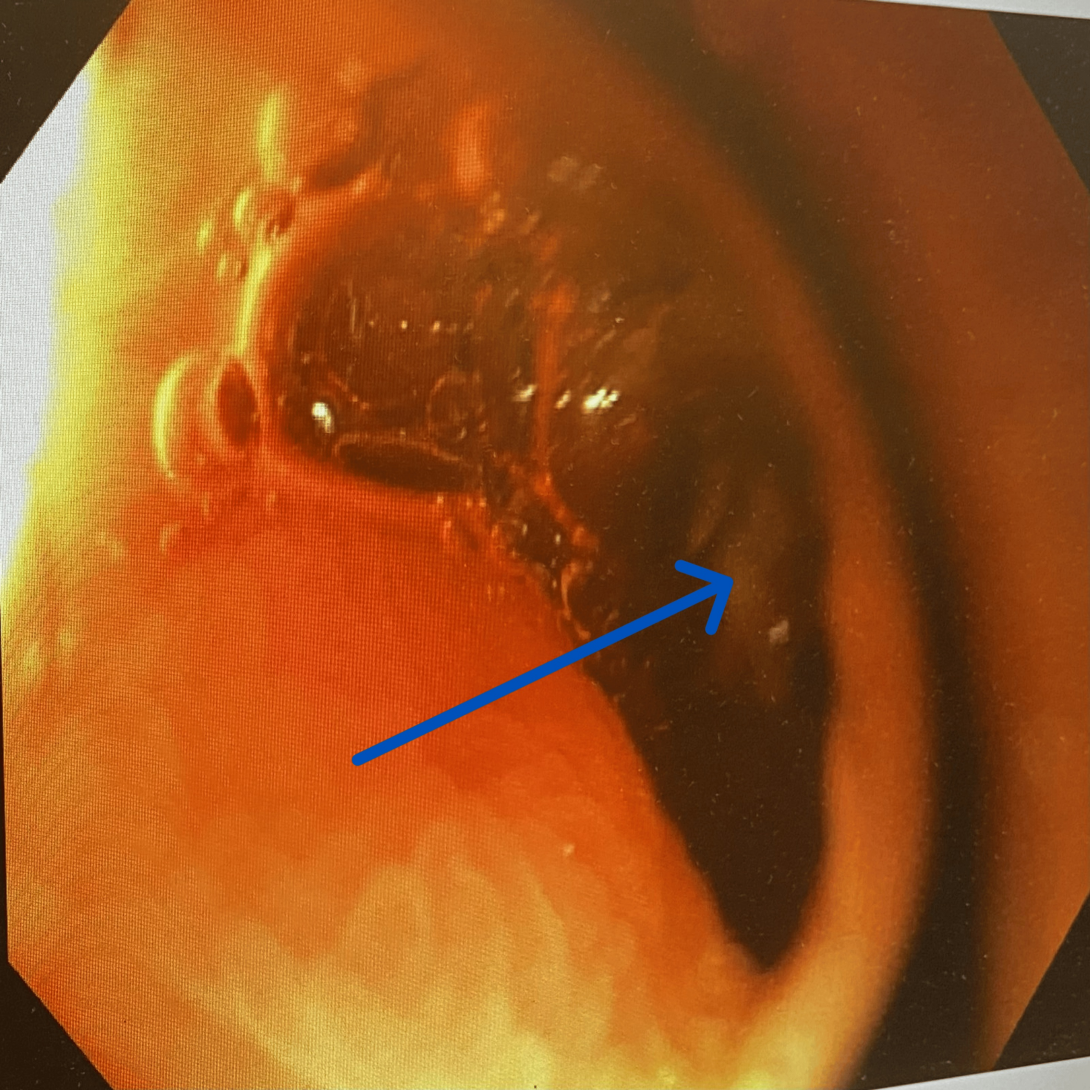
Image showing ulcer at bottom of bulbar diverticulum

**Figure 2:**
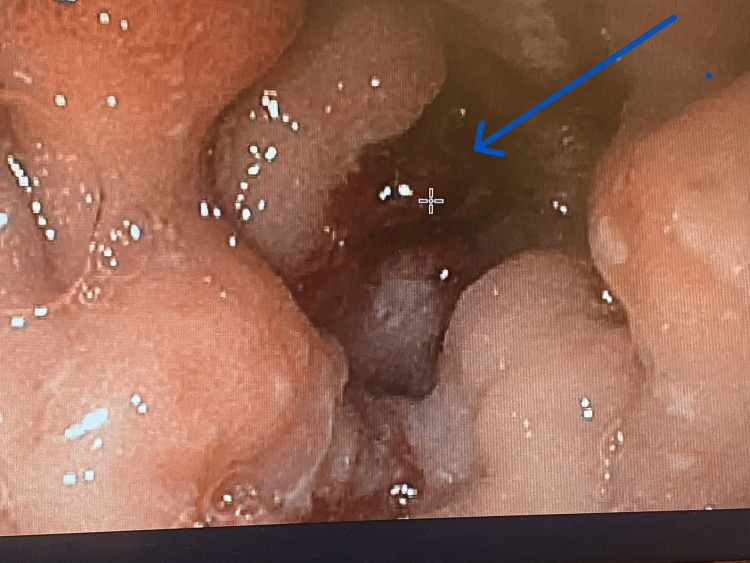
Image showing the non-bleeding visible vessel in the diverticulum (Forrest IIA) in our case

**Figure 3:**
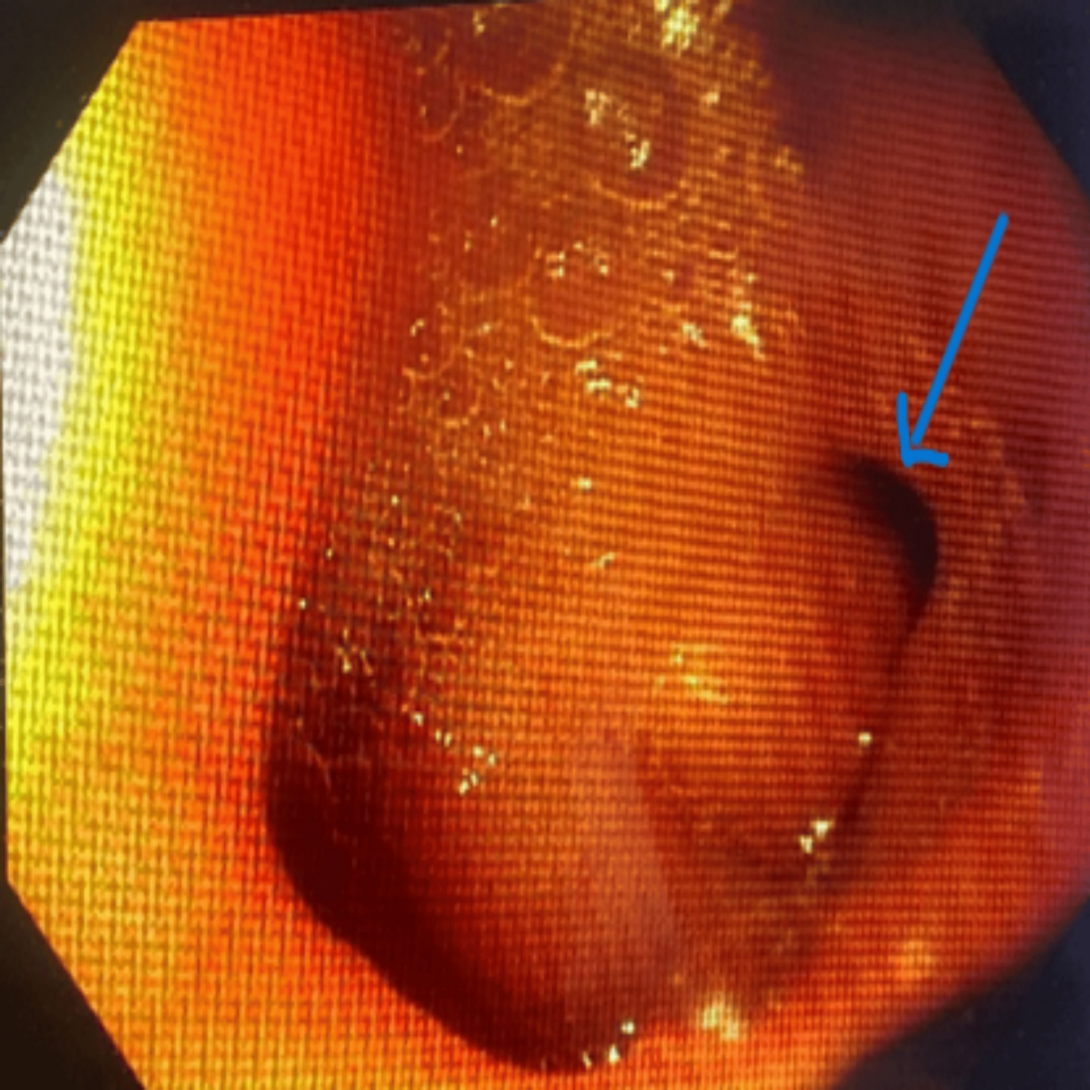
Image showing our patient's ulcerated bulbar diverticulum

This is an 82-year-old man, with no medical history, non-smoker, non-alcoholic. He was admitted to the emergency department for melena (the passage of digested blood through the anus, black and tarry, either mixed with stool or not, and notably very foul-smelling) that had occurred two days prior to admission. Clinically, the patient was hemodynamically and respiratory stable, with a blood pressure of 126/66 mmHg and a heart rate of 61 beats per minute. However, the patient had experienced increasing asthenia (probably related to anemia due to occult bleeding) one week leading up to admission. Laboratory test showed anemia, with a hemoglobin level of 6 g/dL down from 13 g/dL one month prior. The patient received two units of red blood cells, resulting in a post-transfusion hemoglobin level of 8 g/dL. A gastroscopy revealed a crescent-shaped ulcer classified as stage IIa according to the Forrest classification (Figures 1, 2), located within a diverticulum on the anterior wall of the duodenal bulb (Figure 3).

**Figure 4:**
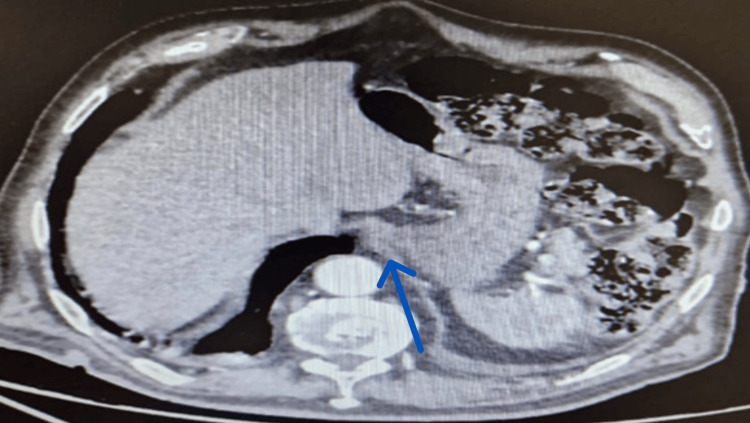
Contrast-enhanced abdominal CT scan does not show any extravasation of the contrast agent or signs of active bleeding

Given the crescentic nature of the ulcer and its diverticular location, endoscopic treatment was deemed risky due to the potential for perforation. Consequently, a medical treatment approach was adopted, along with imaging (abdominal CT) to prepare the patient for potential radiological treatment via embolization in case of recurrence. The contrast-enhanced abdominal CT scan does not show any extravasation of the contrast agent or signs of active bleeding (Figure 4). The patient was placed on intravenous proton pump inhibitors (omeprazole) at a bolus dose of 80 mg, followed by a continuous infusion of 8 mg/h for 72 hours. The patient's condition evolved favorably during hospitalization, with resolution of melena and stabilization of hemoglobin levels, and no recurrence of hemorrhage. Gastric biopsies were performed to investigate for Helicobacter pylori (HP), and the pathological results confirmed an HP infection. An eradication treatment was initiated. Due to the bulbar location of the ulcer, a follow-up gastroscopy was not indicated but no invasive tests for HP specially. The urea breath test was done at the end of the eradication treatment.

## Discussion

The duodenal diverticulum is defined as small sacs formed by folds in the mucous membrane of the intestinal wall or esophagus; it is the second most common site for diverticula after the colon [8]. Most duodenal diverticula are discovered incidentally, with a global prevalence estimated 2% to 5% [9]. This frequency may vary from 2% to 23% according to endoscopy and autopsy studies [10]. Upper gastrointestinal bleeding is a rare but serious complication of duodenal diverticula, unlike colonic diverticula [8] .

According to the literature, ulcer disease, portal hypertension, gastro-duodenal erosions, and reflux esophagitis account for approximately 80% of upper gastrointestinal bleeding [11] ]. In France, despite a decrease in incidence, which is currently about 120 per 100,000 inhabitants per year, gastric and duodenal ulcers remain the leading cause of this type of bleeding and are primarily caused by a pathogenic and carcinogenic bacterium, notably *Helicobacter pylori *infection[12]. In 10% of cases, endoscopy does not allow for the diagnosis of the cause of gastrointestinal bleeding, and in the remaining 10% of cases, the causes are rare, including Mallory-Weiss syndrome, Dieulafoy’s ulcer, pancreatic and biliary hemorrhages, aorto-digestive fistulas, and other causes such as bulbo-duodenal diverticular bleeding [11]. The first-line examination remains gastroscopy to enable targeted endoscopic treatment, reducing morbidity and the risk of recurrent bleeding [13]. Diverticular hemorrhage is a rare and serious complication, and the presence of an ulcer within the diverticula makes it even rarer, with no literature data describing this unusual localization [12]. This highlights the importance of our article presenting this first case. 

In cases of active hemorrhage not related to portal hypertension, several therapeutic options can be considered depending on the etiology, location, and hemodynamic stability of the patient [13]. These options range from medical treatment to endoscopic hemostasis, radiological embolization, and even surgical intervention [13]. If endoscopic treatment is not feasible, an urgent CT scan is essential to identify the source of the bleeding and determine the appropriate treatment as quickly as possible [14]. Emergency endoscopy allows for the diagnosis and treatment of upper gastrointestinal bleeding, ideally performed within the first 24 hours, or sooner if active bleeding is suspected and hemodynamic status permits [14]. It should be conducted by an experienced operator under general anesthesia, with the assistance of trained personnel in endoscopic hemostasis techniques (Table 1) [15,16]. The routine use of a nasogastric tube, considered invasive and of limited utility, is not recommended. It is preferable to administer intravenous erythromycin (250 mg) within 30 minutes prior to the endoscopy to increase the likelihood that the stomach is free of blood [14,15].

**Table 1:** The different methods of endoscopic hemostasis in cases of digestive bleeding outside of portal hypertension [16,17]

Methods	Means	effectiveness	Cost		Indication
Injections	1-Adrenaline (1mg/10 ml); 2-Others: absolute alcohol, polidocanol éthanolamine	Short term		low	-Any active bleeding or with a visible vessel: -Active ulcerative hemorrhage + -Mallory-Weiss lesions with active bleeding -Some situations of failure of other endoscopic hemostasis methods
Thermal	1-Argon plasma coagulation (APC); 2-Others: Bipolar or multipolar coagulation	Proven long-term in inactive ulcerative hemorrhages		average	- Inactive ulcerative hemorrhage -Angiodysplastic lesions
Mechanical	-Hemostatic clips	Proven effectiveness: in cases of ulcerative hemorrhage stage Ia, Ib, IIa, and IIb of Forrest. Low effectiveness in cases of old and fibrous ulcers, tangential presentation or duodenal lesions		high	- Forrest stage Ia to stage IIb ulcer bleeding

In the case of ulcerative hemorrhage, the Forrest classification is well correlated with the risks of recurrent hemorrhage [18]. In the absence of endoscopic treatment, the risks of recurrent hemorrhage for ulcers classified as Forrest Ia (spurting hemorrhage), Ib (oozing), IIa (non-bleeding visible vessel), IIb (adherent clot), IIc (pigmented spots), and III (yellowish-white crater with a clean base) are 90%, 20%, 50%, 20%, 7%, and 3%, respectively [14]. Recurrent ulcerative hemorrhages occur within 72 hours in over 80% of patients. Risk factors for recurrence include the presence of a large ulcer (> 1 cm), active hemorrhage-particularly in Forrest stage Ia during the initial endoscopy-and the ulcer's location (posterior wall of the bulb, upper part of the gastric lesser curvature). In the case of a non-bleeding visible vessel, the risk of recurrent hemorrhage decreases from 50% to 10% following endoscopic treatment [15]. In the event of recurrent hemorrhage, a second endoscopic intervention should be attempted, either using the same hemostatic methods employed during the first endoscopy or by changing techniques, with a success rate of up to 70% [14]. In 95% of cases or more, the initial endoscopic treatment remains successful in cases of digestive hemorrhages due to ulcers [13]. If there is a new recurrence after a second endoscopic treatment, radiological embolization is the preferred treatment [15].

What is the role of medical treatment in cases of ulcer-related hemorrhage?

Numerous studies demonstrate that the administration of high doses of proton pump inhibitors (PPIs) either orally or intravenously, enhances the efficacy of endoscopic treatment, with omeprazole and esomeprazole being the most extensively studied agents [19]. The choice of administration route should be determined by the clinical context, including the severity of the hemorrhage, Forrest classification stage, quality and nature of the hemostatic procedure, and comorbidities. For lesions classified as Forrest Ia or b and IIa or b (which are at highest risk for recurrence), an initial bolus of 80 mg followed by a continuous infusion of 8 mg/h for 72 hours is recommended. In cases of H. pylori infection, PPIs should be administered orally at more standard doses [20]. This is consistent with the initial management of our case. H2-receptor antagonists have not demonstrated efficacy in the treatment of ulcer-related gastrointestinal hemorrhage [20].

## Conclusions

Digestive hemorrhages remain a common emergency that can endanger vital prognosis with a high mortality. This can be explained by sites of hemorrhage that go undetected during gastroscopies. It is therefore crucial to raise awareness among gastroenterologists about the importance of thoroughly exploring gastric diverticula while minimizing insufflation, as this could shed light on our patients' symptoms, particularly regarding gastro-duodenal ulcers, especially in cases of digestive hemorrhage.

## References

[1] Nahon S, Hagège H, Latrive JP (2012). Epidemiological and prognostic factors involved in upper gastrointestinal bleeding: results of a French prospective multicenter study. Endoscopy.

[2] Czernichow P, Hochain P, Nousbaum JB (2000). Epidemiology and course of acute upper gastro-intestinal haemorrhage in four French geographical areas. Eur J Gastroenterol Hepatol.

[3] Costa ND, Cadiot G, Merle C, Jolly D, Bouche O, Thiéfin G, Zeitoun P (2001). Bleeding reflux esophagitis: a prospective 1-year study in a university hospital. Am J Gastroenterol.

[4] Lorenzo D (2021). L’hémorragie digestive en chiffres : qu’avons-nous gagné en 30 ans?. La Presse Médicale Formation.

[5] Elhjouji A, Jaiteh L, Bounaim A, Aitali A, Sair K (2015). [Duodenal diverticulitis: unusual complication not always easy to manage]. Pan Afr Med J.

[6] Bouchentouf S (2008). Les diverticules duodénaux compliqués: éléments diagnostiques et thérapeutiques. À propos de deux cas. JAHG.

[7] Ryan ME, Hamilton JW, Morrissey F (1984). Gastrointestinal hemorrhage from a duodenal diverticulum. Gastrointest Endosc.

[8] Martinez-Cecilia D, Arjona-Sanchez A, Gomez-Alvarez M (2008). Conservative management of perforated duodenal diverticulum: a case report and review of the literature. World J Gastroenterol.

[9] Oukachbi N, Brouzes S (2013). Management of complicated duodenal diverticula. J Visc Surg.

[10] Shuck JM, Stallion A (2001). Duodenal diverticula. Surgical Treatment: Evidence-Based and Problem-Oriented.

[11] Lesur G (2005). Hémorragies digestives hautes de causes rares. Gastroenterol Clin Biol.

[12] Park CH (2004). A prospective, randomized trial of endoscopic band ligation vs. epinephrine injection for actively bleeding Mallory-Weiss syndrome. Gastrointest Endosc.

[13] Romãozinho JM, Pontes JM, Lérias C, Ferreira M, Freitas D (2004). Dieulafoy's lesion: management and long-term outcome. Endoscopy.

[14] Lesur G, Vedrenne B, Heresbach D (2010). Consensus en endoscopie digestive (CED). Acta Endoscopica.

[15] Sung JJ, Barkun A, Kuipers EJ (2009). Intravenous esomeprazole for prevention of recurrent peptic ulcer bleeding: a randomized trial. Ann Intern Med.

[16] Bescós MM, Pontes AR, Muñoz RC (2023). Helicobacter pylori. FMC.

[17] Hooi JK, Lai WY, Ng WK (2017). Global prevalence of Helicobacter pylori infection: systematic review and meta-analysis. Gastroenterology.

[18] Loffroy R (2015). Embolisation artérielle transcathéter dans les hémorragies gastro-intestinales hautes non variqueuses : indications, techniques et résultats. J Radiol Diagn Inter.

[19] Barkun AN, Bardou M, Kuipers EJ, Sung J, Hunt RH, Martel M, Sinclair P (2010). International consensus recommendations on the management of patients with nonvariceal upper gastrointestinal bleeding. Ann Intern Med.

[20] Arora NK, Ganguly S, Mathur P, Ahuja A, Patwari A (2002). Upper gastrointestinal bleeding: etiology and management. Indian J Pediatr.

